# Trends in Mortality Due to Myocardial Infarction, Stroke, and Pulmonary Embolism in Patients Receiving Dialysis

**DOI:** 10.1001/jamanetworkopen.2022.7624

**Published:** 2022-04-18

**Authors:** Gurbey Ocak, Rianne Boenink, Marlies Noordzij, Willem Jan W. Bos, Bjorn E. Vikse, Aleix Cases, Julia Kerschbaum, Jaakko Helve, Maurizio Nordio, Mustafa Arici, Lucile Mercadal, Christoph Wanner, Runolfur Palsson, Kristine Hommel, Johan De Meester, Myrto Kostopoulou, Rafael Santamaria, Emilio Rodrigo, Helena Rydell, Samira Bell, Ziad A. Massy, Kitty J. Jager, Anneke Kramer

**Affiliations:** 1 Department of Internal Medicine, Sint Antonius Hospital, Nieuwegein, the Netherlands; 2 European Renal Association Registry, Department of Medical Informatics, Amsterdam Universitair Medische Centra, University of Amsterdam, Amsterdam Public Health research institute, Amsterdam, the Netherlands; 3 Department of Internal Medicine, Leiden University Medical Center, Leiden, the Netherlands; 4 Department of Clinical Medicine, University of Bergen, Bergen, Norway; 5 Department of Medicine, Haugesund Hospital, Haugesund, Norway; 6 Nephrology Department, Hospital Clínic, Universitat de Barcelona, August Pi i Sunyer Biomedical Research Institute, Barcelona, Spain; 7 Registre de Malalts Renals de Catalunya, Barcelona, Spain; 8 Department of Internal Medicine IV - Nephrology and Hypertension, Austrian Dialysis and Transplant Registry, Medical University Innsbruck, Innsbruck, Austria; 9 Finnish Registry for Kidney Diseases, Helsinki, Finland; 10 Abdominal Center Nephrology, University of Helsinki, Helsinki University Hospital, Helsinki, Finland; 11 Veneto Dialysis and Transplantation Registry, Regional Epidemiology System, Padua, Italy; 12 Nephrology Dialysis and Renal Transplantation Unit, Treviso, Italy; 13 Department of Nephrology, Faculty of Medicine, Hacettepe University, Ankara, Turkey; 14 Department of Nephrology and Renal Transplantation, Assistance Publique-Hôpitaux de Paris, Hôpital de La Pitié Salpêtrière Hospital, Paris, France; 15 Division of Nephrology, Department of Medicine, University Hospital of Würzburg, Würzburg, Germany; 16 Division of Nephrology, Landspitali, The National University Hospital of Iceland, Reykjavik, Iceland; 17 Faculty of Medicine, School of Health Sciences, University of Iceland, Reykjavik, Iceland; 18 Department of Medicine, Holbaek Hospital, Holbaek, Denmark; 19 Department of Nephrology, Dialysis and Hypertension, Dutch-speaking Belgian Renal Registry (NBVN), Sint-Niklaas, Belgium; 20 Department of Nephrology, General Hospital, Athens, Greece; 21 Andalusian Autonomous Transplant Coordination Information System, Seville, Spain; 22 Nephrology ServiceReina Sofia University Hospital, Cordoba, Spain; 23 Nephrology Service, University Hospital Marqués de Valdecilla/IDIVAL, University of Cantabria, Santander, Spain; 24 Division of Renal Medicine, Department of Clinical Sciences Intervention and Technology, Karolinska Institutet, Stockholm, Sweden; 25 Swedish Renal Registry, Department of Internal Medicine, Ryhov Regional Hospital, Jönköping, Sweden; 26 Scottish Renal Registry, Meridian Court, Glasgow, United Kingdom; 27 Division of Population health And Genomics, University of Dundee, Dundee, United Kingdom; 28 Division of Nephrology, Ambroise Paré University Hospital, Boulogne-Billancourt, France; 29 Institut National de la Santé et de la Recherche Médicale, Research Centre in Epidemiology and Population Health, University of Paris Ouest-Versailles-St Quentin-en-Yveline, Villejuif, France

## Abstract

**Importance:**

During the past decades, improvements in the prevention and management of myocardial infarction, stroke, and pulmonary embolism have led to a decline in cardiovascular mortality in the general population. However, it is unknown whether patients receiving dialysis have also benefited from these improvements.

**Objective:**

To assess the mortality rates for myocardial infarction, stroke, and pulmonary embolism in a large cohort of European patients receiving dialysis compared with the general population.

**Design, Setting, and Participants:**

In this cohort study, adult patients who started dialysis between 1998 and 2015 from 11 European countries providing data to the European Renal Association Registry were and followed up for 3 years. Data were analyzed from September 2020 to February 2022.

**Exposures:**

Start of dialysis.

**Main Outcomes and Measures:**

The age- and sex-standardized mortality rate ratios (SMRs) with 95% CIs were calculated by dividing the mortality rates in patients receiving dialysis by the mortality rates in the general population for 3 equal periods (1998-2003, 2004-2009, and 2010-2015).

**Results:**

In total, 220 467 patients receiving dialysis were included in the study. Their median (IQR) age was 68.2 (56.5-76.4) years, and 82 068 patients (37.2%) were female. During follow-up, 83 912 patients died, of whom 7662 (9.1%) died because of myocardial infarction, 5030 (6.0%) died because of stroke, and 435 (0.5%) died because of pulmonary embolism. Between the periods 1998 to 2003 and 2010 to 2015, the SMR of myocardial infarction decreased from 8.1 (95% CI, 7.8-8.3) to 6.8 (95% CI, 6.5-7.1), the SMR of stroke decreased from 7.3 (95% CI, 7.0-7.6) to 5.8 (95% CI, 5.5-6.2), and the SMR of pulmonary embolism decreased from 8.7 (95% CI, 7.6-10.1) to 5.5 (95% CI, 4.5-6.6).

**Conclusions and Relevance:**

In this cohort study of patients receiving dialysis, mortality rates for myocardial infarction, stroke, and pulmonary embolism decreased more over time than in the general population.

## Key Points

**Question:**

What is the trend of mortality rates for myocardial infarction, stroke, and pulmonary embolism in patients receiving dialysis compared with the general population?

**Findings:**

In this cohort study of 220 467 incident patients receiving dialysis, mortality rates decreased over time both in the patients receiving dialysis and in the general population. The adjusted mortality rate ratios decreased between 1998 to 2015 for myocardial infarction, stroke, and pulmonary embolism.

**Meaning:**

Mortality rates for myocardial infarction, stroke, and pulmonary embolism improved more in patients receiving dialysis than in the general population, suggesting possible improvement in predialysis and dialysis care.

## Teaser

This cohort study assesses the mortality rates caused by myocardial infarction, stroke, and pulmonary embolism in a large cohort of European patients receiving dialysis.

## Introduction

Myocardial infarction, stroke, and pulmonary embolism are important cardiovascular causes of death in the general population. During the past decades, substantial improvements have been made in preventing and managing myocardial infarction,^1,2,3,4,5,6,7^ stroke,^8,9,10,11,12,13,14,15^ and pulmonary embolism^16,17,18,19,20,21^ through widespread implementation of the standard of care (eg, statins, renin-angiotensin system inhibitors, antiplatelet drugs, and anticoagulants). Several studies in the general population found decreasing short-term fatality rates after myocardial infarction over time,^21,22,23^ declining hospitalizations and case fatality rates after stroke,^24,25,26^ and decreasing short-term mortality rates after pulmonary embolism.^27,28,29,30,31^

Patients receiving dialysis are at increased risk of myocardial infarction,^32,33^ stroke,^34,35^ and pulmonary embolism.^36,37^ However, it is unknown to what extent they benefited from the improvements in the prevention and management of the aforementioned diseases when compared with the general population. From a clinical perspective, it is important to know whether advances in the prevention and clinical treatment strategies have resulted in improved outcomes among patients receiving dialysis. It is possible that patients receiving dialysis have not achieved significant benefit from these improvements because therapies could be ineffective or underused in this patient group. One example is that in large clinical trials, statin use was not associated with a beneficial effect on cardiovascular outcomes for patients receiving dialysis.^38,39^ On the other hand, dialysis outcomes might have improved because of better dialysis care resulting from enhanced research conducted in patients receiving dialysis. Therefore, this study aimed to investigate the trends in mortality rates for myocardial infarction, stroke, and pulmonary embolism in patients receiving dialysis compared with the general population.

## Methods

### Study Population

The cohort study consisted of incident adult patients receiving dialysis from 11 European countries comprising 8 national and 8 regional kidney registries providing individual patient data to the European Renal Association (ERA) Registry.^40^ Those national and regional registries are from Austria, Dutch- and French-speaking Belgium, Denmark, Finland, Greece, Iceland, the Netherlands, Norway, Scotland, Sweden, and the Spanish regions of Andalusia, Asturias, Basque Country, Catalonia, and Cantabria, and fully cover all patients receiving dialysis within the country or region they are representing. Informed consent was obtained in accordance with national or regional regulations for each registry. Compliance with ethical standards was confirmed by the Medical Ethical Committee of the Amsterdam Medical Centre. We included patients who started dialysis between January 1, 1998, and December 31, 2015, and followed them for a maximum of 3 years until death, censoring for the recovery of kidney function, kidney transplantation, or loss to follow-up, whichever occurred first. This study was reported according to the Strengthening the Reporting of Observational Studies in Epidemiology (STROBE) reporting guideline.

The ERA Registry collects data about patients receiving kidney replacement therapy, including date of birth, sex, primary kidney disease, date of initiation of kidney replacement therapy, and date and cause of death.^41^ Of note, medication use or laboratory data are not included.

### Mortality in Patients Receiving Dialysis

We categorized the causes of death according to the coding system of the ERA Registry, which provides a standardized classification of causes of death in patients receiving kidney replacement therapy.^41^ Mortality because of myocardial infarction was defined as death attributed to myocardial infarction (code 11), mortality because of stroke as death attributed to the cerebrovascular accident (code 22), and mortality because of pulmonary embolism was defined as death attributed to pulmonary embolism (code 21).

### Mortality in the General Population

Mortality data for the general population in the corresponding 11 European countries that contributed data on patients receiving dialysis were used as a reference. These data, derived from the national statistics on causes of death, were obtained from the World Health Organization (WHO).^42^ The WHO reports provide mortality data coded according to the *International Classification of Diseases, Ninth Revision *(*ICD-9*) and *International Statistical Classification of Diseases and Related Health Problems, Tenth Revision *(*ICD-10*) coding system, stratified by age category, sex, and calendar year. Mortality due to myocardial infarction was defined by *ICD-9* codes 410 to 411 and B270 and *ICD-10* codes I21 to I22, mortality due to stroke by *ICD-9* codes 433 to 436 and B292 to B293 and *ICD-10* codes I63 to I64, and mortality because of pulmonary embolism was defined by *ICD-9* codes 415 and B280 and *ICD-10* code I26.

### Statistical Analysis

Continuous variables are presented as median (IQR), and categorical variables are presented as percentages. Mortality rates were calculated by dividing the number of deaths by the total time at risk for both the patients receiving dialysis and the general population. The person-time at risk in the general population of the 11 countries was calculated as the sum of the mean size of the population in the subsequent calendar years using the large-scale demographic method.^43^ The time at risk for the dialysis population was calculated until death or censoring for recovery of renal function, kidney transplantation or loss to follow-up, and maximized to 3 years. Crude mortality rate ratios with 95% CIs were calculated by dividing the mortality rates of patients receiving dialysis by the mortality rates of the general population. Furthermore, age- and sex-adjusted mortality rate ratios with 95% CIs were calculated by dividing the mortality rates of patients receiving dialysis by the mortality rates in the general population of similar age and sex using direct standardization with the dialysis population as reference. In addition, mortality rate ratios with 95% CIs were calculated after stratification for sex and different age categories.

To compare mortality rates over time, time trends were analyzed using joinpoint regression analysis.^44^ This method identifies when a change—a so-called joinpoint—in the trend occurs. To test whether joinpoints were statistically significant and should be added to the model, the Monte Carlo permutation method was used. Changes in the slopes of these trends were calculated as an annual percentage change (APC) with a 95% CI for each segment. In addition, mortality rate ratios with 95% CIs were calculated for 3 equal and predefined periods (ie, 1998-2003, 2004-2009, and 2010-2015).

All analyses were performed using SPSS statistical software version 23.0 (SPSS) or SAS statistical software version 9.4 (SAS Institute), except for the joinpoint regression analyses, for which we used Joinpoint software version 4.7.0.0 (National Cancer Institute). Data were collected from January 1998 to January 2016 and analyzed from September 2020 to February 2022.

## Results

### Baseline Characteristics

In total, 220 467 patients receiving dialysis from 11 European countries who started dialysis between 1998 and 2015 were included in the study. Their median age was 68 (56.5-76.4) years, and 82 068 patients (37.2%) were female (Table 1). The percentage of male patients receiving dialysis increased from 40 412 (61.0%) in the 1998 to 2003 period to 49 852 (64.4%) in the 2010 to 2015 period. Diabetes was the most common cause of kidney failure (52 879 [24.0%]). For the total observation period, the median duration of follow-up in patients receiving dialysis was 2.4 years, with a total observation time of 436 389 person-years. During the observation period, 49 955 (22.7%) of the patients underwent kidney transplantation. The data set contained no missing values for age or sex. For 39 842 patients (18.1%), the primary kidney disease was missing and the cause of death for 7823 patients (3.5%) (Table 1).

**Table 1.** Baseline Characteristics of Patients Receiving Dialysis

Characteristic	Patients, No. (%)			
	All (N = 220 467)	1998-2003 (n = 66 242)	2004-2009 (n = 76 827)	2010-2015 (n = 77 398)
Age, median (IQR), y	68.2 (56.5-76.4)	66.5 (54.2-74.5)	68.6 (56.9-76.7)	69.3 (58.1-77.7)
Sex				
Female	82 068 (37.2)	25 830 (39.0)	28 692 (37.3)	27 546 (35.6)
Male	138 399 (62.8)	40 412 (61.0)	48 135 (62.7)	49 852 (64.4)
Primary kidney disease				
Polycystic kidney disease	12 346 (5.6)	3960 (6.0)	4237 (5.5)	4149 (5.4)
Pyelonephritis	12 376 (5.6)	4434 (6.7)	4310 (5.6)	3632 (4.7)
Glomerulonephritis	25 218 (11.4)	8747 (13.2)	8440 (11.0)	8031 (10.4)
Hypertension	26 351 (12.0)	7240 (10.9)	9099 (11.8)	10 012 (12.9)
Renal vascular disease	12 737 (5.8)	3724 (5.6)	5238 (6.8)	3775 (4.9)
Diabetes	52 879 (24.0)	15 297 (23.1)	18 927 (24.6)	18 655 (24.1)
Multisystem disease	18 336 (8.3)	5841 (8.8)	6207 (8.1)	6288 (8.1)
Miscellaneous	20 382 (9.2)	5567 (8.4)	6500 (8.5)	8315 (10.7)
Unknown	39 842 (18.1)	11 432 (17.3)	13 869 (18.1)	14 541 (18.8)
Treatment modality				
Hemodialysis	185 912 (84.3)	55 264 (83.4)	64 999 (84.6)	65 649 (84.8)
Peritoneal dialysis	34 555 (15.7)	10 978 (16.6)	11 828 (15.4)	11 749 (15.2)
Kidney transplantation at the end of follow-up (within 3 y)	49 955 (22.7)	16 539 (25.0)	17 288 (22.5)	16 128 (20.8)
Follow-up time on dialysis, median (IQR), y	2.4 (0.9-3.0)	2.3 (0.9-3.0)	2.4 (0.9-3.0)	2.5 (1.0-3.0)

The general population yielded a total observation time of 1327 million person-years. The age and sex distribution of the general population and patients receiving dialysis are shown in eFigure 1 in the Supplement. In the general population, 51.4% of person-years were attributable to female individuals, and 8.1% were aged 20 to 24 years of age, 18.0% were aged 25 to 34 years, 19.3% were aged 35 to 44 years, 18.0% were aged 45 to 54 years, 15.0% were aged 55 to 64 years, 11.6% were aged 65 to 74 years, 7.5% were aged 75 to 84 years, and 2.5% were aged 85 years or older.

### Occurrence of Deaths for Myocardial Infarction, Stroke, and Pulmonary Embolism

During follow-up, 83 912 of the 220 467 patients receiving dialysis died (Table 2). Myocardial infarction was the cause of death in 7662 cases (9.1%), stroke in 5030 cases (6.0%), and pulmonary embolism in 435 cases (0.5%). In the general population, 15 835 391 persons died during the study period. Of these, 1 183 903 people (7.5%) died from myocardial infarction, 82 705 people (6.1%) died from stroke, and 965 065 people (0.5%) died from pulmonary embolism. The proportion of myocardial infarction, stroke, and pulmonary embolism as the cause of death was lower from 2010 to 2015 than in the earlier periods both in the dialysis and general population (Table 2).

**Table 2.** Causes of Death in Patients Receiving Dialysis and the General Population

Cause of death	Patients, No. (%)			
	All	1998-2003	2004-2009	2010-2015
Patients receiving dialysis				
Total deaths	83 912 (100)	26 505 (100)	29 708 (100)	27 699 (100)
Myocardial infarction	7662 (9.1)	3044 (11.5)	2730 (9.2)	1888 (6.8)
Stroke	5030 (6.0)	1898 (7.2)	1750 (5.9)	1382 (5.0)
Pulmonary embolism	435 (0.5)	182 (0.7)	149 (0.5)	104 (0.4)
Other	70 785 (84.4)	21 381 (80.7)	25 079 (84.4)	24 325 (87.8)
General population				
Total deaths	15 835 391 (100)	5 266 933 (100)	5 200 622 (100)	5 367 835 (100)
Myocardial infarction	1 183 903 (7.5)	501 542 (9.5)	388 169 (7.5)	294 192 (5.5)
Stroke	82 705 (6.1)	31 918 (7.5)	27 327 (6.0)	23 461 (4.8)
Pulmonary embolism	965 065 (0.5)	393 711 (0.6)	312 401 (0.5)	258 953 (0.4)
Other	13 603 718 (85.9)	4 339 762 (82.4)	4 472 725 (86.0)	4 791 229 (89.3)

### Time Trends in Mortality Rates of Myocardial Infarction

Crude mortality rates for myocardial infarction decreased by 4.0% (95% CI, 2.7% to 5.3%) per year from 1998 to 2007 and by 6.9% (95% CI, 8.4% to 5.4%) per year from 2007 to 2015 in patients receiving dialysis (eFigure 2 in the Supplement). There was also a decline in mortality rates for myocardial infarction during the study in the general population (annual percentage change [APC], −3.8%; 95% CI, −5.0% to −2.5%) from 1998 to 2002 and APC −5.3% (95% CI, −5.5% to −5.1%) from 2003 to 2015 (eFigure 2 in the Supplement). The age- and sex-standardized mortality rate ratios for myocardial infarction in patients receiving dialysis as compared with the general population decreased from 8.1 (95% CI, 7.8 to 8.3) between 1998 and 2003 to 6.8 (95% CI, 6.5 to 7.1) between 2010 and 2015 (Table 3).

**Table 3.** Mortality Rates in Patients Receiving Dialysis and the General Population

Condition	Time periods			
	All	1998-2003	2004-2009	2010-2015
Myocardial infarction				
Mortality rate for patients receiving dialysis^a^	17.6	23.7	17.9	12.2
Mortality rate for general population^a^	0.9	1.2	0.9	0.6
Crude mortality rate ratio (95% CI)	19.7 (19.2-20.1)	19.9 (19.2-20.6)	20.4 (19.7-21.2)	19.1 (18.3-20.0)
Age- and sex-standardized mortality rate ratio (95% CI)	7.2 (7.1-7.4)	8.1 (7.8-8.3)	7.4 (7.1-7.6)	6.8 (6.5-7.1)
Stroke				
Mortality rate for patients receiving dialysis^a^	11.5	14.8	11.5	8.9
Mortality rate for general population^a^	0.7	0.9	0.7	0.6
Crude mortality rate ratio (95% CI)	15.9 (15.4-16.3)	15.8 (15.1-16.5)	16.3 (15.5-17.1)	15.9 (15.1-16.7)
Age- and sex-standardized mortality rate ratio (95% CI)	6.2 (6.1-6.4)	7.3 (7.0-7.6)	6.2 (5.9-6.5)	5.8 (5.5-6.2)
Pulmonary embolism				
Mortality rate for patients receiving dialysis^a^	1.0	1.4	1.0	0.7
Mortality rate for general population^a^	0.1	0.1	0.1	0.1
Crude mortality rate ratio (95% CI)	16.0 (14.6-17.6)	18.7 (16.2-21.6)	15.8 (13.5-18.6)	13.2 (10.9-16.0)
Age- and sex-standardized mortality rate ratio (95% CI)	6.8 (6.2-7.5)	8.7 (7.6-10.1)	6.6 (5.6-7.7)	5.5 (4.5-6.6)
Other				
Mortality rate for patients receiving dialysis^a^	162.2	166.2	164.3	156.8
Mortality rate for general population^a^	10.2	10.3	10.1	10.4
Crude mortality rate ratio (95% CI)	15.8 (15.7-15.9)	16.2 (15.9-16.4)	16.3 (16.1-16.5)	15.1 (14.9-15.3)
Age- and sex-standardized mortality rate ratio (95% CI)	6.3(6.3-6.3)	7.3 (7.2-7.4)	6.3 (6.3-6.4)	5.7 (5.6-5.7)

^a^ Mortality rates are expressed as deaths per 1000 person-years.

### Time Trends in Mortality Rates of Stroke

The trends in crude mortality rates for stroke were similar to myocardial infarction. The mortality decreased during the study in both the dialysis population (APC, −3.9%; 95% CI, −4.5% to −3.3%) and the general population (APC, −4.1%; 95% CI, −4.3% to −3.9%) (eFigure 3 in the Supplement). The age- and sex-standardized mortality rate ratios for stroke also decreased over time from 7.3 (95% CI, 7.0% to 7.6%) between 1998 and 2003 to 5.8 (95% CI, 5.5% to 6.2%) between 2010 and 2015 (Table 3).

### Time Trends in Mortality Rates of Pulmonary Embolism

Similar to myocardial infarction and stroke, there was a decline in mortality rates of pulmonary embolism throughout the study period in patients receiving dialysis (APC, −5.6%; 95% CI, −7.2%) and in the general population (APC −3.2%; 95% CI, −3.4% to −2.9%) (eFigure 4 in the Supplement). The related age- and sex-standardized mortality rate ratios decreased from 8.7 (95% CI, 7.6 to 10.1) between 1998 and 2003 to 5.5 (95% CI, 4.5 to 6.6) between 2010 and 2015 (Table 3).

### Time Trends in Mortality Rates of Other Causes of Death

The trends in mortality rates for other causes were slightly different from the trends for myocardial infarction, stroke, and pulmonary embolism (eFigure 5 in the Supplement). In patients receiving dialysis, no decrease in mortality rates of other causes of death was observed between 1998 and 2003 (APC, 1.0%; 95% CI, −0.3 to 2.3), while there was a decline between 2003 and 2015 (APC, −0.9%; 95% CI, −1.2 to −0.5). In the general population, the APC was −0.3% (95% CI, −0.5 to 0) from 1998 to 2009 and 1.0% (95% CI, 0.3% to 1.7%) in 2009 to 2014. However, the age- and sex-standardized mortality rate ratios showed a similar pattern for other causes of death as the mortality rate ratios for myocardial infarction, stroke, and pulmonary embolism; the ratios decreased from 7.3 (95% CI, 7.2 to 7.4) between 1998 and 2003 to 5.7 (95% CI, 5.6 to 5.7) between 2010 and 2015 (Table 3).

### Time Trends in Mortality Rate Ratios Stratified for Sex and Age

Although mortality rate ratios of myocardial infarction, stroke, pulmonary embolism, and other causes of death were consistently higher among females compared with males, age- and sex-standardized mortality rate ratios decreased over time for both females and males (Table 4). Mortality rate ratios of myocardial infarction, stroke, pulmonary embolism, and other causes of death decreased consistently over time in patients aged 65 years or above (Table 5).

**Table 4.** Mortality Rate Ratios in Patients Receiving Dialysis and the General Population Stratified by Sex and Time Period

Condition	Mortality rate ratio (95% CI)			
	All	1998-2003	2004-2009	2010-2015
Myocardial infarction				
Female patients				
Crude	19.7 (18.9-20.5)	19.6 (18.4-20.9)	20.5 (19.1-21.7)	18.6 (17.1-20.2)
Age standardized	8.9 (8.5-9.2)	10.1 (9.5-10.7)	9.0 (8.4-9.6)	7.9 (7.2-8.6)
Male patients				
Crude	18.4 (17.9-18.9)	18.9 (18.1-19.8)	19.2 (18.3-20.1)	17.9 (16.9-18.9)
Age standardized	6.7 (6.5-6.9)	7.3 (7.0-7.7)	6.8 (6.5-7.1)	6.4 (6.1-6.8)
Stroke				
Female patients				
Crude	15.3 (14.7-16.0)	14.5 (13.6-15.6)	16.0 (14.9-17.1)	15.8 (14.6-17.1)
Age standardized	7.1 (6.8-7.4)	8.2 (7.6-8.8)	7.1 (6.7-7.7)	6.6 (6.1-7.1)
Male patients				
Crude	18.2 (17.5-18.9)	18.7 (17.7-19.9)	18.4 (17.3-19.6)	18.0 (16.8-19.3)
Age standardized	5.7 (5.5-5.9)	6.8 (6.4-7.2)	5.6 (5.3-6.0)	5.4 (5.0-5.8)
Pulmonary embolism				
Female patients				
Crude	16.5 (14.3-19.0)	18.1 (14.6-22.6)	18.6 (14.9-23.3)	11.6 (8.5-16.0)
Age standardized	7.6 (6.6-8.8)	9.6 (7.7-11.9)	8.3 (6.6-10.4)	5.1 (3.7-7.0)
Male patients				
Crude	17.1 (15.1-19.4)	20.8 (17.1-25.3)	15.1 (11.9-19.0)	15.8 (12.4-20.1)
Age standardized	6.3 (5.5-7.1)	8.2 (6.7-9.9)	5.4 (4.3-6.8)	5.7 (4.5-7.3)
Other				
Female patients				
Crude	16.3 (16.1-16.5)	16.8 (16.5-17.2)	16.8 (16.5-17.2)	15.4 (15.0-15.7)
Age standardized	7.9 (7.8-8.0)	9.5 (9.3-9.7)	7.9 (7.8-8.1)	6.8 (6.7-6.9)
Male patients				
Crude	15.4 (15.3-15.5)	15.6 (15.3-15.8)	15.8 (15.6-16.1)	14.9 (14.6-15.1)
Age standardized	5.6 (5.6-5.7)	6.3 (6.2-6.4)	5.7 (5.6-5.8)	5.2 (5.1-5.3)

**Table 5.** Mortality Rate Ratios in Patients Receiving Dialysis and the General Population Stratified by Age and Time Period

Condition and age group	Mortality rate ratio (95% CI)			
	All	1998-2003	2004-2009	2010-2015
Myocardial infarction				
20-44 y				
Crude	84.7 (70.4-101.9)	108.6 (85.5-137.8)	69.8 (49.3-98.7)	48.0 (27.9-82.8)
Age and sex standardized	53.8 (44.7-64.7)	68.8 (54.2-87.2)	44.7 (31.6-63.2)	30.3 (17.6-52.1)
45-64 y				
Unstandardized	28.6 (27.2-30.1)	27.6 (25.6-29.8)	29.1 (26.7-31.6)	28.2 (25.4-31.3)
Age and sex standardized	21.0 (19.9-22.0)	20.5 (19.0-22.2)	21.1 (19.4-23.0)	20.4 (18.4-22.6)
≥65 y				
Unstandardized	6.7 (6.5-6.9)	6.8 (6.5-7.1)	6.9 (6.6-7.2)	6.7 (6.3-7.0)
Age and sex standardized	6.1 (6.0-6.3)	6.7 (6.5-7.0)	6.3 (6.0-6.5)	5.8 (5.5-6.1)
Stroke				
20-44 y				
Crude	713.6 (601.1-847.2)	835.4 (660.5-1056.5)	614.2 (454.1-830.8)	537.1 (340.0-848.5)
Age and sex standardized	539.7 (456.5-638.1)	642.1 (511.2-806.4)	466.9 (347.5-627.4)	386.1 (246.2-605.3)
45-64 y				
Unstandardized	83.6 (78.2-89.3)	76.9 (69.3-85.3)	82.8 (73.9-92.8)	92.2 (80.8-105.3)
Age and sex standardized	62.5 (58.5-66.7)	58.1 (52.4-64.4)	61.7 (55.2-69.1)	68.0 (59.7-77.4)
≥65 y				
Unstandardized	4.8 (4.6-4.9)	4.7 (4.5-5.0)	4.8 (4.6-5.1)	4.9 (4.6-5.2)
Age and sex standardized	5.0 (4.9-5.2)	5.8 (5.5-6.1)	5.1 (4.8-5.3)	4.9 (4.6-5.2)
Pulmonary embolism				
20-44 y				
Crude	58.3 (33.1-102.8)	59.7 (24.8-143.8)	57.2 (21.4-152.7)	56.0 (18.0-174.1)
Age and sex standardized	47.9 (27.2-84.3)	49.1 (20.4-118.0)	47.3 (17.7-126)	45.5 (14.7-141.0)
45-64 y				
Unstandardized	29.4 (24.2-35.9)	30.9 (22.6-42.1)	30.5 (21.8-42.5)	25.4 (17.0-38.0)
Age and sex standardized	25.3 (20.8-30.8)	26.5 (19.5-36.2)	26.1 (18.7-36.4)	21.8 (14.6-32.6)
≥65 y				
Unstandardized	5.2 (4.7-5.8)	6.3 (5.3-7.4)	5.0 (4.1-6.0)	4.3 (3.4-5.4)
Age and sex standardized	5.4 (4.9-6.0)	7.1 (6.0-8.4)	5.2 (4.3-6.2)	4.3 (3.5-5.4)
Other				
20-44 y				
Crude	43.8 (41.6-46.1)	41.7 (38.4-45.2)	42.7 (39.1-46.6)	47.4 (42.9-52.4)
Age and sex standardized	35.7 (33.9-37.6)	34.1 (31.4-37.0)	34.9 (32.0-38.1)	38.3 (34.7-42.3)
45-64 y				
Unstandardized	20.4 (20.0-20.7)	20.6 (20.0-21.3)	20.2 (19.6-20.9)	20.0 (19.4-20.7)
Age and sex standardized	16.5 (16.2-16.8)	16.9 (16.4-17.4)	16.4 (15.9-16.9)	16.0 (15.5-16.6)
≥65 y				
Unstandardized	5.6 (5.6-5.7)	5.8 (5.7-5.9)	5.7 (5.7-5.8)	5.4 (5.4-5.5)
Age and sex standardized	5.5 (5.4-5.5)	6.2 (6.1-6.3)	5.5 (5.5-5.6)	5.0 (4.9-5.1)

## Discussion

In this cohort study of incident patients receiving dialysis, we observed that mortality rates for myocardial infarction, stroke, and pulmonary embolism decreased between 1998 and 2015. In the general population, mortality rates for myocardial infarction, stroke, and pulmonary embolism were also lower for the 2010 to 2015 period than the 1998 to 2010 period. After adjustment for age and sex, the reduction in the mortality rates of myocardial infarction, stroke, and pulmonary embolism in the dialysis population was greater than in the general population. In patients aged 65 years or older, the mortality decreased consistently over time for the different cardiovascular causes of death.

Only a limited number of studies have investigated trends in mortality for myocardial infarction, stroke, and pulmonary embolism in patients receiving dialysis. A previous study using data from the United States Renal Data System showed that in-hospital mortality rates decreased over time for myocardial infarction (31.9% in 1993 and 18.8% in 2008) and 2-year cumulative probability of death after admission for myocardial infarction (76.5% in 1993 and 71.5% in 2008) in patients receiving dialysis.^45^ Furthermore, a single-center study in the Netherlands showed a 3-fold decrease in 30-day myocardial infarction fatality rate in patients with chronic kidney disease stages 4 and 5 between 2000 and 2008 compared with the 1985 to 1990 period.^46^ However, in these studies, mortality rates in patients receiving dialysis were not compared with those in the general population. Studies have been lacking since 2008. Using data from the Nationwide Inpatient Sample in the United States, a previous study investigated stroke incidence and fatality rates in dialysis and non-patients receiving dialysis.^47^ The results showed that in-hospital mortality was significantly higher in the dialysis group than in the nondialysis group and that in the 2003 to 2014 period, the differences in mortality between these groups decreased over time. During the study, the in-hospital mortality declined from 11% to 5% in the dialysis group and from 6% to 4% in the nondialysis group. These findings are in line with those of our study. General population studies from France,^30^ Italy,^31^and the United States^27^ have shown a reduction in mortality rates because of pulmonary embolism. This is consistent with our findings. To our knowledge, the current study is the first to investigate trends in fatal pulmonary embolism in patients receiving dialysis compared with the general population.

There are several potential reasons for the decreasing mortality rate ratios due to myocardial infarction, stroke, and pulmonary embolism in patients receiving dialysis compared with the general population. In the last decades, many improvements have been made in the prevention and management of myocardial infarction^1,2,3,4,5,6,7^ (eg, the use of antiplatelet drugs, including clopidogrel, prasugrel, and ticagrelor; β-blockers; renin-angiotensin system inhibitors; statins; and implantable cardioverter defibrillators), stroke^8,9,10,11,12,13,14,15^ (eg, the use of dipyridamole, clopidogrel, carotid endarterectomy, and statins), and pulmonary embolism^16,17,18,19^ (eg, the use of vena cava filters, low-molecular-weight heparin use for patients who are hospitalized, and the use of computed tomographic angiography). A reason may be that recent advances in the prevention and management of these disorders are more beneficial for patients receiving dialysis than in the general population. However, this is not in line with the results of most studies on management aimed at reducing cardiovascular events, which have not shown benefit in patients receiving dialysis, while a clear improvement has previously been demonstrated in the general population. For example, 2 large trials of statin use did not demonstrate a beneficial effect on cardiovascular outcomes for patients receiving dialysis.^38,39^ Additionally, it was shown that antiplatelet treatment in patients with chronic kidney disease administered in addition to standard care in persons with acute coronary syndromes or those undergoing percutaneous coronary revascularization had little or no effect on the incidence of myocardial infarction, death, or coronary revascularization.^48^ Furthermore, the effect of vitamin K antagonist therapy for the prevention of stroke in patients receiving dialysis with atrial fibrillation is debatable.^49,50,51,52^ Other potential reasons for the decreasing mortality rate ratios for myocardial infarction, stroke, and pulmonary embolism may include better management of acute cardiovascular events in patients receiving dialysis, changes in dialysis setting (eg, the introduction of dialysis quality measures and standardization of dialysis processes) and better management of dialysis-specific risk factors for mortality, including electrolyte disorders, anemia, bone mineral disorders, and hypotensive episodes. Furthermore, the care of patients with chronic kidney disease could be more effective than before, leading to better preservation of health (fewer comorbidities) at the start of dialysis, which may have contributed to a decrease in cardiovascular risk. A previous analysis of the ERA Registry data showed a decrease in the prevalence of cardiovascular comorbidities at the onset of the dialysis population over time.^53^ The decrease in mortality rate ratios for other causes of death could be an argument in favor of both improved predialysis care and a better treatment of dialysis-specific risk factors for mortality. This finding agrees with a recent study showing a decrease in excess all-cause mortality risk in the dialysis population compared with the general population.^54^ Finally, more conservative care (without dialysis) in elderly patients with kidney failure in recent years may have introduced selection bias. Consequently, dialysis may have been started by elderly patients who were healthier, which may have resulted in decreasing mortality rates over time.

Although mortality rates declined, clinicians should still be aware of the poorer outcomes of myocardial infarction, stroke, and pulmonary embolism for patients receiving dialysis compared with the general population. Prevention and management schemes that are effective in the general population could not be beneficial for patients receiving dialysis. Therefore, in our opinion, future studies should focus on tailored therapy for patients receiving dialysis rather than on implementing prevention and treatment schemes that are recommended for the general population. A major strength of this study is the large number of patients receiving dialysis in this population-based international cohort with available information on causes of death.

### Limitations

This study has limitations. First, information on comorbidities, medication use, and laboratory data was unavailable. Therefore, we were unable to investigate the association between these important factors and trends in mortality for myocardial infarction, stroke, and pulmonary embolism, and consequently, residual confounding may remain. Second, we had no data on baseline estimated glomerular filtration rate to investigate whether changes in dialysis initiation could have influenced our results. Third, we had no information about nonfatal events of myocardial infarction, stroke, or pulmonary embolism. Therefore, we could not investigate the trends for nonfatal events. Fourth, the methods for assigning the cause of death in the dialysis and general population differed. Causes of death in patients receiving dialysis are usually recorded by the nephrologist, whereas causes of death within the general population are, according to law, recorded by the physician who confirmed the death. This may have introduced bias in the calculation of the rate ratios between the dialysis population and the general population. However, we do not think that the practices of physicians determining the cause of death differed over time. Therefore, this is unlikely to have influenced our trend analyses. Fifth, the causes of death were not validated. Although the accuracy of the data is probably high because the nephrologists who treated these patients recorded the causes of death, we cannot rule out misclassification. Nevertheless, we have no reason to believe that this coding accuracy may have changed over time. Sixth, no comparisons were made between countries, but existing differences are unlikely to have changed significantly over time.

## Conclusions

Our results suggest that age- and sex-standardized mortality rate ratios for myocardial infarction, stroke, and pulmonary embolism in patients receiving dialysis decreased over time compared with the general population. This improvement may be due to better predialysis and dialysis care.

## Notes

Supplement. **eFigure 1.** Age and Sex Distribution of Patients Receiving Dialysis and the General Population **eFigure 2.** Incidence of Fatal Myocardial Infarction in Patients Receiving Dialysis and the General Population **eFigure 3.** Incidence of Fatal Stroke in Patients Receiving Dialysis and the General Population **eFigure 4.** Incidence of Fatal Pulmonary Embolism in Patients Receiving Dialysis and the General Population **eFigure 5.** Incidence of Causes of Death Other Than Myocardial Infarction, Stroke and Pulmonary Embolism in Patients Receiving Dialysis and the General Population
