# Supplementary material for: Trends in Mortality Due to Myocardial Infarction, Stroke, and Pulmonary Embolism in Patients Receiving Dialysis
Source: JAMA Netw Open. 2022 Apr 18;5(4):e227624. doi: 10.1001/jamanetworkopen.2022.7624 (PMC9016490; doi:10.1001/jamanetworkopen.2022.7624)
Supplement: Supplement. — eFigure 1. Age and Sex Distribution of Patients Receiving Dialysis and the General Population eFigure 2. Incidence of Fatal Myocardial Infarction in Patients Receiving Dialysis and the General Population eFigure 3. Incidence of Fatal Stroke in Patients Receiving Dialysis and the General Population eFigure 4. Incidence of Fatal Pulmonary Embolism in Patients Receiving Dialysis and the General Population eFigure 5. Incidence of Causes of Death Other Than Myocardial Infarction, Stroke and Pulmonary Embolism in Patients Receiving Dialysis and the General Population [file jamanetwopen-e227624-s001.pdf]

# Supplemental Online Content

Ocak G, Boenink R, Noordzij M, et al. Trends in mortality due to myocardial infarction, stroke, and pulmonary embolism in patients receiving dialysis. *JAMA Netw Open*. 2022;5(4):e227624. doi:10.1001/jamanetworkopen.2022.7624

**eFigure 1.** Age and Sex Distribution of Patients Receiving Dialysis and the General Population

**eFigure 2.** Incidence of Fatal Myocardial Infarction in Patients Receiving Dialysis and the General Population

**eFigure 3.** Incidence of Fatal Stroke in Patients Receiving Dialysis and the General Population

**eFigure 4.** Incidence of Fatal Pulmonary Embolism in Patients Receiving Dialysis and the General Population

**eFigure 5.** Incidence of Causes of Death Other Than Myocardial Infarction, Stroke and Pulmonary Embolism in Patients Receiving Dialysis and the General Population

This supplemental material has been provided by the authors to give readers additional information about their work.

© 2022 Ocak G et al. *JAMA Network Open.*

eFigure 1. Age and Sex Distribution of Patients Receiving Dialysis and the General Population

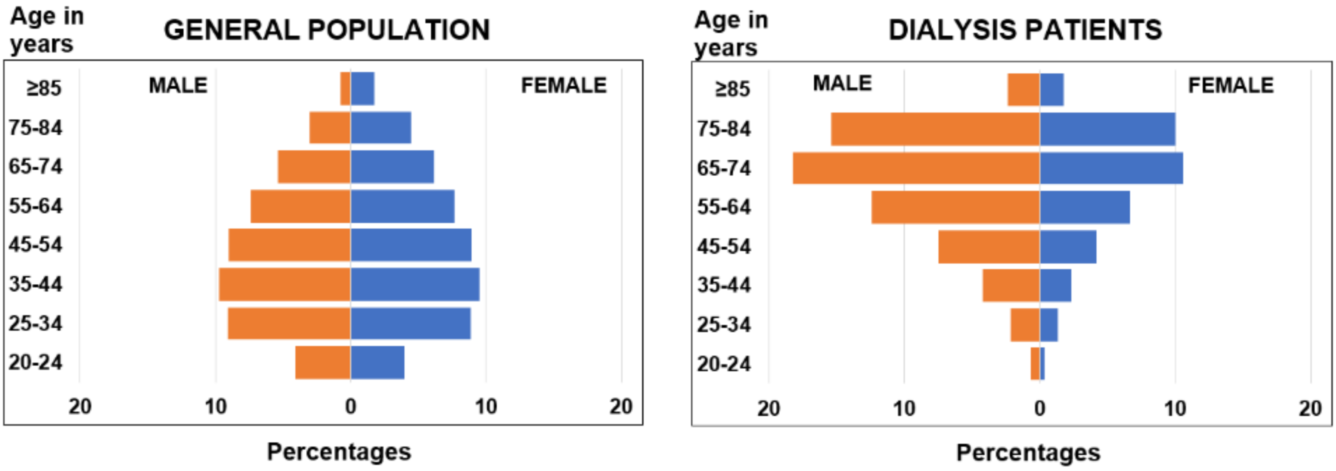


© 2022 Ocak G et al. *JAMA Network Open.*

eFigure 2. Incidence of Fatal Myocardial Infarction in Patients Receiving Dialysis and the General Population

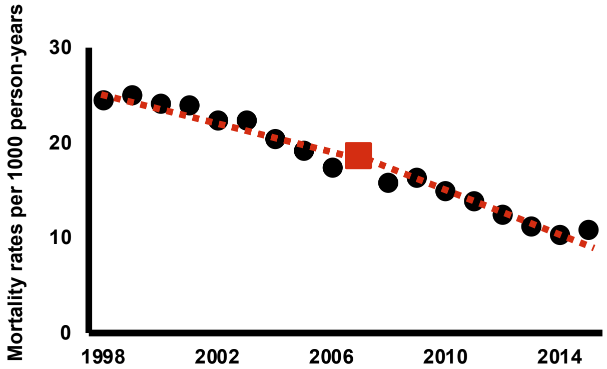


## DIALYSIS PATIENTS

| PERIOD | ANNUAL PERCENTAGE CHANGE |
| --- | --- |
| 1998-2007 | -4.0 (95% CI -5.3; -2.7) |
| 2007-2015 | -6.9 (95% CI -8.4; -5.4) |

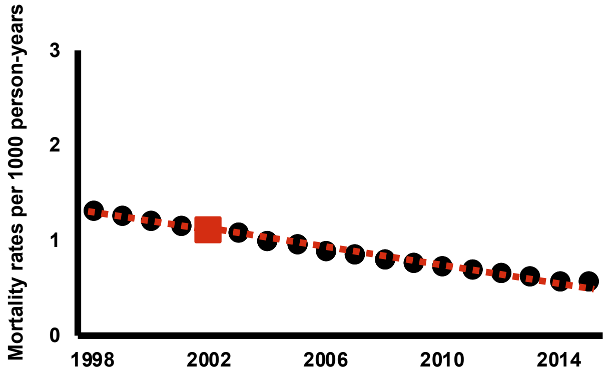


## GENERAL POPULATION

| PERIOD | ANNUAL PERCENTAGE CHANGE |
| --- | --- |
| 1998-2002 | -3.8 (95% CI -5.0; -2.5) |
| 2002-2015 | -5.3 (95% CI -5.5; -5.1) |

Squares represent joinpoints.

© 2022 Ocak G et al. *JAMA Network Open.*

eFigure 3. Incidence of Fatal Stroke in Patients Receiving Dialysis and the General Population

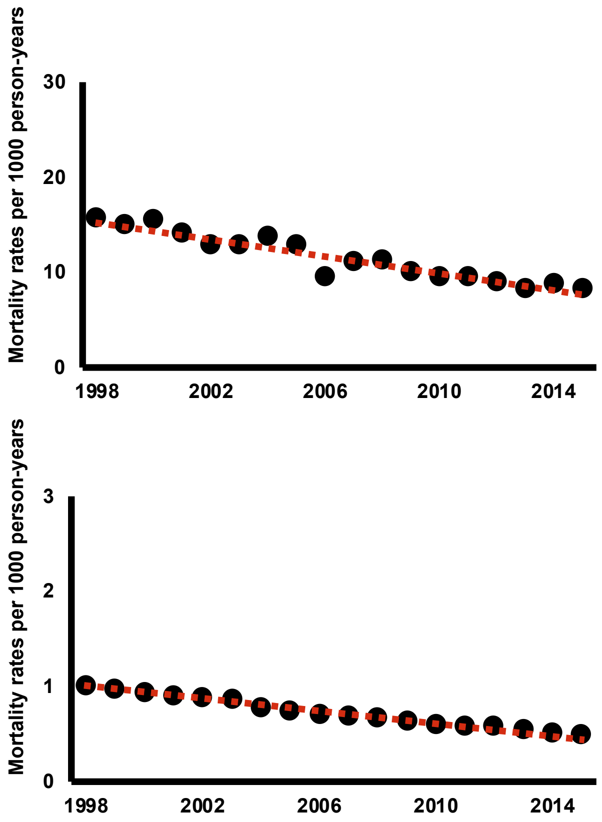


**DIALYSIS PATIENTS**

| PERIOD | ANNUAL PERCENTAGE CHANGE |
|---|---|
| 1998-2015 | -3.9 (95% CI -4.5; -3.3) |

**GENERAL POPULATION**

| PERIOD | ANNUAL PERCENTAGE CHANGE |
|---|---|
| 1998-2015 | -4.1 (95% CI -4.3; -3.9) |

© 2022 Ocak G et al. *JAMA Network Open.*

eFigure 4. Incidence of Fatal Pulmonary Embolism in Patients Receiving Dialysis and the General Population

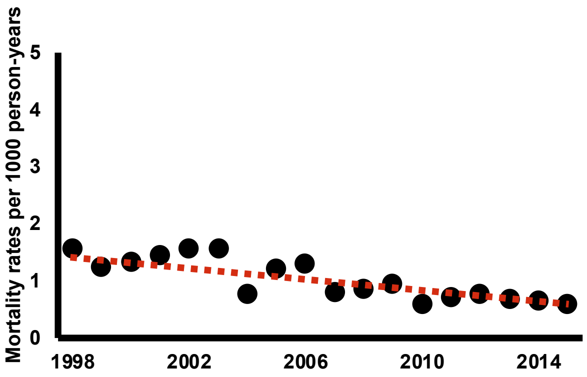


**DIALYSIS PATIENTS**

| PERIOD | ANNUAL PERCENTAGE CHANGE |
|---|---|
| 1998-2015 | -5.6 (95% CI -7.2; -3.9) |

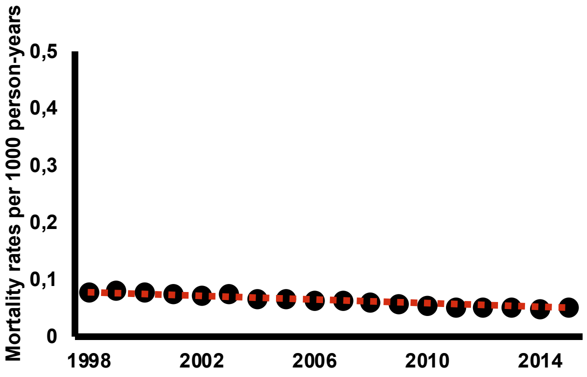


**GENERAL POPULATION**

| PERIOD | ANNUAL PERCENTAGE CHANGE |
|---|---|
| 1998-2015 | -3.2 (95% CI -3.4; -2.9) |

© 2022 Ocak G et al. *JAMA Network Open.*

eFigure 5. Incidence of Causes of Death Other Than Myocardial Infarction, Stroke and Pulmonary Embolism in Patients Receiving Dialysis and the General Population

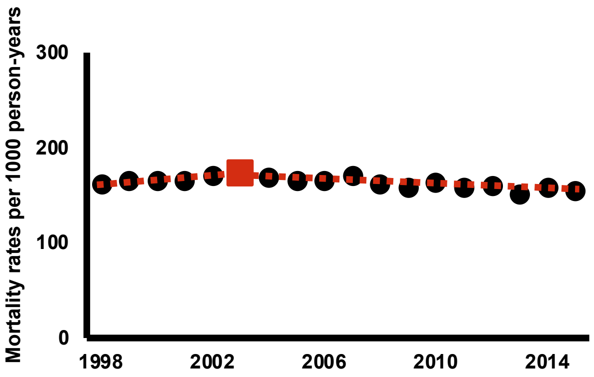


**DIALYSIS PATIENTS**

| PERIOD | ANNUAL PERCENTAGE CHANGE |
|---|---|
| 1998-2003 | 1.0 (95% CI -0.3; 2.3) |
| 2003-2015 | -0.9 (95% CI -1.2; -0.5) |

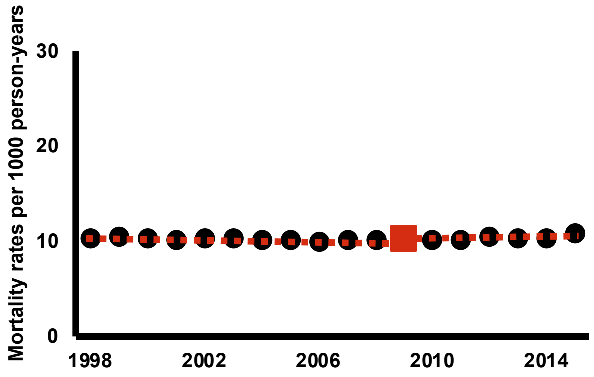


**GENERAL POPULATION**

| PERIOD | ANNUAL PERCENTAGE CHANGE |
|---|---|
| 1998-2009 | -0.3 (95% CI -0.5; 0.0) |
| 2009-2015 | 1.0 (95% CI 0.3; 1.7) |

Squares represent joinpoints.

© 2022 Ocak G et al. *JAMA Network Open.*
